# Prevalence of depression and associated factors among HIV/AIDS patients attending antiretroviral therapy clinic at Dessie referral hospital, South Wollo, Ethiopia

**DOI:** 10.1186/s13033-020-00389-0

**Published:** 2020-07-29

**Authors:** Seada Seid, Oumer Abdu, Mebratu Mitiku, Koku Sisay Tamirat

**Affiliations:** 1 Amhara Regional Health Bureau, Borumeda Hospital, Dessie, Ethiopia; 2 Department of Internal Medicine, College of Medicine and Health Science, University of Gondar, Gondar, Ethiopia; 3 Department of Epidemiology and Biostatistics, Institute of Public Health, College of Medicine and Health Science, University of Gondar, Gondar, Ethiopia

**Keywords:** Depression, HIV/AIDS, Dessie, Ethiopia

## Abstract

**Introduction:**

Depression is one of the common mental health disorders and predicted to be the second cause of the global health burden by the year 2020. Depression in HIV patients may lead to poor engagement to their HIV care which may finally result in poor treatment outcomes. Therefore, the aim of this study was to assess the prevalence of depression and associated factors among HIV/AIDS patients on ART at Dessie referral hospital.

**Methods:**

An institution based cross-sectional study was conducted among 395 HIV positive adult patients on antiretroviral treatment from November to January 2019. The study participants were selected by using the systematic random sampling technique among patients who visited the antiretroviral (ART) clinic in the hospital and standardized Patients Health Questionnaire (PHQ-9) was used to measure depression. Descriptive statistics like percentage, median with interquartile range (IQR) was computed and presented in the form of text and table. Binary logistic regression model was fitted to identify factors associated with depression. An adjusted odds ratio (AOR) with a 95% confidence interval (CI) was used to identify factors associated with depression.

**Result:**

The prevalence of depression was found to be 20% with (95% CI 16.2, 23.8). Age group of 25–34 years (AOR = 6.58, 95% CI 1.11, 38.9), widowed marital status (AOR = 7.05, 95% CI 2.32, 21.38), perceived stigma (AOR = 2.43, 95% CI 1.13, 5.21)], had opportunistic infections [AOR = 4.96, 95% CI (1.05, 23.34)], HIV non-disclosed HIV status (AOR = 6.34, 95% CI 1.34–29.65), poor and fair drug adherence (AOR = 7.1, 95% CI 2.06, 24.44), CD4 count ≤ 200 (AOR = 5.38, 95% CI 2.37–12.23) were factors significantly associated with depression.

**Conclusion:**

The magnitude of depression was relatively lower than the pooled estimates for Ethiopia. Perceived stigma, younger age, widowed, being symptomatic, fair and poor adherence, recent opportunistic infection, low CD4 count, and HIV status not disclosed were positively associated with depression. This finding suggests the integration of mental health care with antiretroviral therapy and the special emphasis ought to be given for those at higher risk of depression.

## Background

Mental health problems accounted for 13% of the global burden of disease and are highly intertwined with infectious diseases like HIV/AIDS [1, 2]. Depression is mental health disorder which is characterized by depressed mood, loss of interest or pleasure, decreased energy, feelings of guilt or low self-worth, disturbed sleep or appetite and poor concentration [3, 4]. Currently, an estimated 350 million peoples are affected by depression worldwide and predicted to be the second most important cause of the global disease burden by the year 2020 [3, 4]. However, in low- and middle-income countries treatment and support services for depression are often absent or underdeveloped. An estimated 76–85% of people suffering from mental disorders in these countries lack access to the treatment they need.

According to the global HIV/AIDS 2017 report, 36.7 million people were estimated to live with HIV/AIDS. About two-thirds of the HIV infected people in the world were located in sub-Saharan Africa and around 43% of new cases were in Eastern and Southern Africa including Ethiopia [5]. Ethiopia is one of the sub-Saharan countries highly affected by HIV, though the prevalence decreased from 1.5% in 2011 to 1.16% in 2017, it is still a great public health problem [6, 7]. Due stigmatizing nature of the disease depressive disorder was three times higher among HIV infected individuals, the lifetime prevalence of depression range from 22 to 45% [8, 9] compared to those HIV negative which is 3 to 17% [10]. Depression associated with different unfavorable health outcomes among HIVAIDS patients like, increased suicidal attempt, hopelessness, poor drug adherence, fast disease progression, drug resistance, treatment failure and decreased quality of life of patients. This may lead to hospitalization and the increasing cost of medical care.

Depression among HIV patients is related to a combination of clinical and socio-demographic factors. Some of clinical factors might be relatively unique to HIV patient, such as AIDS-related stigma [11], compromised immune status (low CD4 counts) and opportunistic infections [12]; on the other hand socio-demographic factors including gender, low educational level and unemployment have been associated with depression in both HIV negative and positive populations [13].

However, most health professionals fail to recognize depression and other mental problems among HIV/AIDS patients due to lack of regular screening and integrated services. There are limited studies that assessed the magnitude and predictors of depression among HIV patients. Moreover, the available didn’t address important clinical characteristics that might be associated with depression.

Therefore, this study aimed to assess the prevalence and associated factors of depression among HIV/AIDS patients. Findings from this study could help clinicians for appropriate patient management. In addition, it could be also useful as entry point for the integration of mental health care services with antiretroviral treatment.

## Methods

### Study design, setting and period

An institution-based cross-sectional study was conducted from November to January, 2019, at Dessie referral hospital. The hospital is found in the Amhara region located 401 km far from Addis Ababa, the capital city of Ethiopia. The hospital is serving more than five million people of the town and nearby districts. In addition to the general services, the hospital also provides antiretroviral therapy services for more than 6, 763 HIV/AIDS (6279 adults and 484 pediatric) patients since the service started.

### Population and samples

Patients aged 18 years and above and took ART treatment for at least 6 months and attending ART clinic during the data collection period were the study population.

The sample size was determined by using single proportion formula with the assumptions of prevalence (P) of depression among HIVAIDS positive adults (38.94%) obtained from a study conducted in Debrebrhan [14] hospital, 95% confidence level, margin of error 5%, and 10% non-response rate and the final sample size became 403. Finally, the study participants selected by using systematic random sampling technique among ART clinic visitors.

### Data collection procedure

Before initiation of the data collection process questionnaire was prepared in English by reviewing different literatures then translated to local language Amharic to keep its consistencies. The data collection tool has socio demographic, clinical, stigma, psychosocial and depression measurement questions. Depression was measured using the nine-item standardized Patient Health Questionnaire (PHQ-9) depressive symptoms scale. It has a Likert scale with the following response options: 0 = not at all, 1 = several days, 2 = more than half the days, and 3 = nearly every day. It has 0–27 scoring as minimal (1–4), mild (5–9), moderate (10–14), moderately severe (15–19) and severe depressive symptoms (20–27). This is one of the most widely used scales and has been also used in Ethiopia, it is reliable, valid, sensitive and specific to measure depression [15].

Data were collected by interview administered questionnaire to recall for depressive symptoms within 2 weeks, other socio-demographic and clinical characteristics harvested through patient document review. To ensure the validity of the study pretest of the tool and 2 days training was given for two data collectors and one supervisor about the objective of the study and how to make and interview with HIV/AIDS patients.

Depression: Participants who were interviewed and scored five and above using patient health questioner 9 (PHQ-9) were considered as depressed [15].

Good adherence: If the average adherence level (95% or more adherence = missing ≤ 2 doses of 30 doses or ≤ 3 doses of 60 doses) and try to assess by asking the missed doses with in the past 3 days, within 1 week and within 1 month [16].

Fair adherence : (85–94% or 4–8 doses missed per month) [16].

Poor adherence: (less than 85% or ≥ 9 doses missed per month) [16].

Perceived stigma: Participants who scored above the median score ≥ 24 were perceived stigma and who scored below the median score (< 24) were not perceived stigma measured by using the HIV stigma scale [17].

Social support: The OSS-3 scores ranged from 3 to 14 with a score of 3–8 = poor support; 9–11 = moderate support; and 12–14 = strong support [18].

Body Mass Index: based on Nutritional assessment classification underweight < 18.5 kg/m2, Normal = 18.5–24.9 kg/m2, over weight ≥ 25 kg/m2 [19].

### Data processing and analysis

The data was cleaned and entered into Epi info version 7 then exported to STATA Version 14 for data management and analysis. Descriptive statistics like percentage, median (IQR) were used to summarize socio-demographic and clinical characteristics and presented in the form of text, table and graphs. Binary logistic regression model was fitted to identify factors associated with depression on HIV/AIDS patients. Crude and adjusted odds ratio with 95% CI computed to see the presence and strength of association between independent variables and depression. Variables having a p-value of 0.05 and less in the multivariable logistic model considered as significantly associated with depression. Model fitness was checked by using Hosmer and Lemeshow goodness of fit test.

### Ethical consideration

Ethical clearance was obtained from the University of Gondar College of medicine and health sciences ethical review committee before data collection process. An official permission letter was also written to Dessie referral hospital. Respondents were informed about the purpose of the study and verbal consent was obtained from each respondent. Moreover, all the study participants were informed that they have a full right to decline from the study and they were also assured for an attainment of confidentiality for the information obtained from them. Patients who had depression during data collection were linked to psychiatry clinic for further treatment and intervention.

## Results

### Socio demographic characteristics

In this study, a total of 395 HIV/AIDS patients were included with the response rate of 93.5%. The median age of the respondents was 38 [interquartile range (IQR): 10] years and about 46.9% aged between 35 and 44 years. Two hundred forty-two (61.3%) of participants were females, 43.3% were married and about 52.9% had no formal education. Most of the respondents 357 (90.4%) were urban dwellers and similarly 80.3% were living with their families (Table 1).

**Table 1:** Socio demographic characteristics of HIV positive patients in Dessie referral hospital, South Wollo, Ethiopia (n = 395)

Characteristics	Category	Frequency(n)	Percentage (%)
Age	18–24	13	3.3
	25–34	114	28.8
	35–44	182	46.1
	45–54	58	14.6
	≥ 55	28	7
Sex	Male	153	38.7
	Female	242	61.3
Residence	Urban	357	90.4
	Rural	38	9.6
Marital status	Married	176	44.5
	Single	74	18.7
	Divorced	60	15.3
	Widowed	85	21.5
Level of education	No formal education	209	52.9
	Primary school	93	23.5
	Secondary school	60	15.2
	Diploma and above	33	8.3
Occupation	Unemployed	18	4.5
	Government employed	69	17.5
	Housewife	144	36.5
	Daily laborer	99	25.1
	Merchant	37	9.3
	Farmers	28	7.1
Income in ETB	< 2000	234	59.2
	≥ 2000	161	40.8
With whom you live	Rented accommodation	186	47.1
	Own home	209	52.9

### Clinical characteristics of the respondents

The majority (88.4%) of the respondents were on first line ART regimen, of which TDF-3TC-EFV (34.7%) and AZT-3TC-NVP (22%) regimens were commonly used combinations. The majority (83.5%) of the respondents were at recent WHO clinical stage I, of whom most (81.3%) of them had CD4 count of > 200 cells/mm^3^ and about (92.2%) patients had been on ART for more than 12 months. Regarding to ART adherence, the majority (92.4%) of the participants had a good drug adherence, 91.9% had disclosed their HIV status and 45.3% have faced stigma (Table 2).

**Table 2:** Clinical and behavioral characteristics of study participants at Dessie referral hospital, South Wollo, Ethiopia, 2019 (n = 395)

Characteristics	Category	Frequency (n)	Percentage (%)
ART regimen	First line	349	88.4
	Second line	46	11.6
Duration since known to have HIV	≤ 12	24	6.1
	> 12	371	93.9
Recent CD4 count	≤ 200	74	18.7
	> 200	321	81.3
Recent viral load	Not detected	279	70.6
	Up to 999	31	7.9
	> 1000	85	21.5
Hgb level	< 12	55	13.9
	≥ 12	340	86.1
WHO T stage	Stage I	330	83.5
	Stage II and above	37	9.4
	Stage III	23	5.8
	Stage IV	5	1.3
Recent adherence to HHART	Good	365	92.4
	Fair and poor	30	7.6
Opportunistic infections	Yes	31	7.8
	No	364	92.2
Recent hospitalization	Yes	21	5.3
	No	364	94.7
Stigma	Yes	179	45.3
	No	216	54.7
Disclosed HIV status	Yes	374	94.7
	No	21	5.3
Cigarette smoking	Yes	6	1.5
	No	389	98.5
Khat chewing	Yes	38	9.6
	No	357	90.4
Alcohol intake	Yes	17	4.3
	No	378	95.7
Suicidal ideation	Yes	32	8.1
	No	363	91.9
Suicidal attempt	Yes	6	1.5
	No	389	98.5
Social support	Poor	223	56.4
	Moderate	158	40
	Strong	14	3.5

### Prevalence of depression

The prevalence of depression was found to be 20% with (95% CI 16.2, 23.8). Regarding to the severity of depression, 51.9%, 39.2%, 7.6% and 1.3% had mild, moderate, moderately sever and severe depression, respectively. The prevalence of depression among male and female patients was found to be 18.3% and 21.07%, respectively.

### Factors associated with depression

In the multivariable logistic regression analysis, age group of 25–34 year, widowed marital status, perceived stigma, CD4 count ≤ 200 cells/mm^3^, symptomatic patients (WHO stage II and above), non-disclosed HIV status, recent opportunistic infections and fair and poor drug adherence were significantly associated with depression.

As a result, those participants who were widowed and age group of 25–34 year, the odds of depression was 7.05 (AOR = 7.05, 95% CI 2.32, 21.38) and 6.58 (AOR = 6.58, 95% CI 1.11, 38.95) times higher compared single ones and those of older than 55 year, respectively. Similarly, symptomatic patients and those with CD4 count ≤ 200, the odds of depression was 2.51 [AOR = 2.51, 95% CI (1.38, 4.56)] and 5.94 [AOR = 5.94, 95% CI (2.68, 13.62)] times higher compared to those who were clinically stable and had higher CD4 count, respectively. Likewise, patients who experienced stigma and didn’t disclosed their HIV status, the odds of depression was 2.28 [AOR = 2.28, 95% CI (1.12, 4.94)] and 5.38 [AOR = 5.38, 95% CI (1.083, 26.73)] times higher compared to their counter parts. Recent opportunistic infections also associated with 5.18 times higher odds of depression compared to those who had no such history [AOR = 5.18, 95% CI (1.12, 24.55)]. Furthermore, those who had fair and poor drug adherence, the odds of depression was six times higher compared to those good adherence [AOR = 5.96, 95% CI (1.74, 20.52)] (Table 3).

**Table 3:** Factors associated with depression among HIV/AIDS patients on ART at Dessie referral hospital, South Wollo, Ethiopia, 2019 (n = 395)

Category	Depression		COR (95% CI)	AOR (95% CI)
	Yes	No		
Age				
18–24	5	8	2.29 (.412, 13.98)	1.53 (0.1, 22.66)
25–34	37	77	1.76 (1.12, 13.96)	6.58 (1.11, 38.95) *
35–44	25	157	0.58 (0.43, 5.44)	1.35 (.251, 7.27)
45–54	6	52	0.42 (0.22, 4.08)	1.01 (.144, 7.17)
≥ 55	6	22	1	1
Marital status				
Married	9	167	1	1
Single	20	54	6.87 (2.95, 15.99)	3.33 (0.98, 18.9.4)
Divorced	8	52	2.86 (1.05, 7.78)	0.766 (0.22, 2.66)
Widowed	42	43	18.12 (8.19–40.10)	7.05 (2.32, 21.38) *
Perceived stigma				
Yes	50	129	2.45 (1.502, 4.16)	2.43 (1.13, 5.21) *
No	29	187	1	1
Suicidal ideation				
Yes	23	13	9.57 (4.58, 20.01)	4.33 (0.98, 16.28)
No	56	303	1	1
Adherence				
Fair and poor	22	8	14.86 (6.31, 35.01)	7.1 (2.14, 24.44) *
Good	57	308	1	1
Opportunistic infection				
Yes	18	13	6.88 (3.20, 14.77)	4.96 (1.05, 23.34) *
No	61	303	1	1
Recent hospitalization				
Yes	15	6	12.11 (4.52, 32.4)	1.63 (0.27, 9.55)
No	64	310	1	1
Disclosure				
No	15	6	12.11 (4.52, 32.4)	6.34 (1.36, 29.65) *
Yes	64	310	1	1
Social support				
Poor	56	167	2.01 (.44, 9.27)	0.67 (0.07, 6.43)
Moderate	21	137	0.92 (0.19, 4.4)	0.34 (0.03, 3.33)
Strong	2	12	1	1
WHO T stage				
Stage I	55	275	1	1
Stage II and above	24	41	2.93 (1.64, 5.24)	0.84 (.26, 2.63) *
Recent CD4 count				
≤ 200	37	37	6.64 (3.79, 11.62)	5.38 (2.37, 12.23) *
> 200	42	279	1	1

* Show a p-value less than 0.05

## Discussion

This study showed that the prevalence of depression was 20% with 95% CI (16.2, 23.8). This finding is higher than studies done in Ethiopia in different places Debremarkos (11.7%) [11] and Axum (14.6%) [20]. On the other hand this finding is lower than studies done in other parts of Ethiopia, which ranged from 38.4% to 76.7% [14, 21–29]. The prevalence of depression in this study is similarly lower when compared to a systematic review and meta-analysis of east Africa studies (38%) [30], and another pooled estimates in Ethiopia (36.65%) [31] and a systemic review in China 60% [32]. This discrepancy might be due to the variation in sample size, the population being studied, the study period, inclusion criteria and measurement tools used to assess depression. For instance in the study done in Bahirdar depression was measured using the hospital anxiety and depression scale (HADS), whereas a study conducted in Tigray depression was measured by using the 21 items Hamilton’s depression scale Questionnaire. In addition, most of our study participants were clinically stable and most took ART for at least 6 months where immunological recovery achieved.

This study showed that the odds of depression was higher among younger age group patients compared to elders. This could be due to the fact that HIV/AIDS is stigmatizing disease that affects psychosocial well-being of patients. Similarly, this study also revealed that widowed marital status was associated with increased occurrence of depression compared to those married. This finding was supported by previous studies in Ethiopia (16). Death of marital partner and loved one associated with pathological grief and bereavement leads to sadness and loneliness that could increase depression [33].

Perceived stigma associated with increased occurrence of depression compared to those who didn’t experience stigma. This result is consistent with those of previous studies done in Ethiopia [22, 24–26, 29] and Uganda [13]. Perceived stigma go along with low self-image, social isolation and ultimately steered to develop psychological distress and depression [34].

Immunocompromised condition reflected by low CD4 count and those who are symptomatic were associated depression occurrence compared to those clinically stable and had high CD4 count. This result is consistent with studies done in Hawassa, Ethiopia [26] and Malawi [35]. Similarly, a study conducted in Uganda showed that CD4 count > 200 was associated with lower occurrence of depression (protective) [13]. Similarly a study conducted in Ethiopia Yekatit 12 hospital [28] showed that CD4 count < 250 was associated with depression and similar finding in Cameron showed that CD4 count < 100 was associated with high depression [6]. Besides systematic review done in Greece showed that symptomatic patients were more depressed than non-symptomatic patients [36].

In addition, this study also showed that recent opportunistic infections were associated with increased occurrence of depression. Opportunistic infections often associated with hospitalization and diminished functional status which could affect patient’s psychosocial and economic conditions. This result is in agreement with a study done in Alert hospital, Ethiopia [25].

Moreover, patient who were not strictly adherent to their antiretroviral medication were more depressed than those in good adherence. Poor adherence may cause instable clinical condition and loosen patient-physician relationship, ultimately leads to diminished concentration, and feelings of worthlessness and disrupt self-management activities. However, due the cross-sectional nature of the study poor adherence might be the result of depression which is the limitation of the study and difficult to establish cause-effect relationship. This finding is in agreement with a study done in Ethiopia [25, 12, 20, 28, 29] and Korea [37].

Furthermore, non-disclosed HIV status was associated with increased depression occurrence compared to those who disclosed their HIV status. This result is consistent with a study done in Dilla, Ethiopia [38].

This study had strengths of assessing the burden of mental health problems among HIV/AIDS which could be helpful for program consideration and service integration. However, institutional and cross-sectional nature of the study may limit the cause and effect and affect representativeness of the findings. In addition, there may be recall bias from respondents.

## Conclusion

The magnitude of depression was relatively lower than pooled estimates of Ethiopia. Perceived stigma, widowed marital status, being symptomatic, fair and poor adherence, recent opportunistic infection, low CD4 count and non-disclosed HIV status were associated with depression. This finding suggests that depression is a huge problem on HIV patients. Thus integrating mental health care with antiretroviral therapy might be helpful. Special emphasis ought to be given for those at higher risk of depression.

## Data Availability

The datasets used during the current study is available from the corresponding author on reasonable request.

## Abbreviations

AIDS: Acquired Immunodeficiency Syndrome
AOR: Adjusted odds ratio
ART: Antiretroviral therapy
ARVs: Anti Retro Virus
BMI: Body Mass Index
CD4: Cluster of cell Differentiation 4
CI: Confidence interval
COR: Crude Odds Ratio
FMOH: Federal Ministry of Health
HAAR: Highly Active Anti Retro Viral Therapy
HIV: Human immunodeficiency virus
IQR: Inter quartile range
NACP: National Acquired immunodeficiency Syndrome Control Program
OI: Opportunistic infections
PHQ-9: Patients Health Questionnaire
PLWHA: People living with HIV
UNAIDS: United Nations Program on Acquired Immune Deficiency Syndrome
WHO: World Health Organization
